# The grafts modified by heparinization and catalytic nitric oxide generation used for vascular implantation in rats

**DOI:** 10.1093/rb/rby003

**Published:** 2018-03-06

**Authors:** Jingchen Gao, Li Jiang, Qinge Liang, Jie Shi, Ding Hou, Di Tang, Siyuan Chen, Deling Kong, Shufang Wang

**Affiliations:** State Key Laboratory of Medicinal Chemical Biology, Key Laboratory of Bioactive Materials for Ministry of Education, College of Life Sciences, Nankai University, Tianjin 300071, China

**Keywords:** vascular grafts, heparin, nitric oxide, macrophage polarization

## Abstract

Small-diameter (<6 mm) vascular grafts are increasingly needed in peripheral vascular surgery but have few successes because of acute thrombosis, incomplete endothelialization and intimal hyperplasia after implantation. This study used electrospun poly(ε-caprolactone) as the matrix material. Heparin and selenium-containing catalyst-organoselenium modified polyethyleneimine were introduced through layer-by-layer assembly in order to build a vascular graft with *in situ* nitric oxide (NO) generation. The aim of this study was to explore the application of the graft with improved histocompatibility and biological function for vascular implantation in rats. After implantation in rats, compared to poly(ε-caprolactone), the modified grafts could promote the adhesion and proliferation of endothelial cells, and inhibit the adhesion of smooth muscle cells. The modified grafts remarkably promoted endothelialization, inhibited intimal hyperplasia and increased the ratio of alternatively activated macrophages (M2) to classical activated macrophages (M1). This work constructed a vascular graft with heparinization and catalytic NO generation for improving the vascularization, and accelerating the tissue regeneration by regulating the inflammatory response. The present study indicates that it is a promising method for regulating response and tissue regeneration of small diameter vascular grafts by a novel approach of combining heparinization and catalytic NO generation.

## Introduction

Vascular diseases, specifically cardiovascular disease, are the number one cause of death globally [1, 2]. Segments of autologous vessels represent the gold standard and have demonstrated superior clinical performance; however, autografts are limited in supply and dimensions, and so not always available [3, 4]. Meantime, synthetic grafts perform poorly in small-diameter vascular grafts because of acute thrombosis and intimal hyperplasia (IH) after implantation [5, 6]. Thus, there is a substantial need for small-diameter vascular grafts as bypass and blood vessel replacement.

The ideal vascular graft should have the ability to promote endothelialization, inhibit IH and enhance the regeneration of tissue. Endothelialization is important for artificial vascular grafts, because it can provide an appropriate compatible surface to guarantee blood flow and control smooth muscle cell (SMC) proliferation [7]. Selective promotion of endothelial cells (ECs) in competitive adhesion against SMCs can get a balanced behavior of ECs and SMCs for reduced thrombosis and IH [8, 9].

Electrospinning is an efficient process to manufacture grafts for tissue engineering, because electrospun grafts can provide a favorable microenvironment similar to the natural extracellular matrix for cell adhesion and proliferation [10]. Poly(ε-caprolactone) (PCL) is a commonly used candidate material for electrospun grafts applied in artificial blood vessels, because of its good mechanical properties, low cost and high stability in processing and storage. However, as a synthetic polyester, PCL lacks the ability to induce cell proliferation and migration because of its hydrophobic and bio-inert properties [11]. Layer-by-layer (LBL) self-assembly is an easy and versatile technique, which is adaptable for the modification of PCL, and the introduction of bioactive molecules onto the surface of PCL would enable the materials catalyzing ability and bioactivity. In the present study, heparinization and nitric oxide (NO) generation were chosen to promoting endothelialization and inhibit IH.

Heparin is a widely used and reliable anticoagulant and has also been widely used in vascular grafts because of its ability to inhibit migration and proliferation of SMCs [12–16]. Previous studies have proved that an appropriate heparin dosage selectively enhances EC but inhibits SMC proliferation, which means that the surface with an adequate heparin density will inhibit thrombosis and restenosis but not harm the endothelium [7, 14, 17]. In addition, heparin was also shown to exhibit excellent anti-inflammatory performance in various inflammatory disease models [18]. NO has the ability to reduce inflammation and IH, and promote EC growth [19, 20]. There are various endogenous NO donors in the peripheral blood, such as S-nitrosothiols (RSNOs (S-nitrosoglutathione (GSNO), S-nitrosoalbumin (AlbSNO), S-nitrosocysteine (CysSNO) and so on)), and the release of NO from RSNOs can be accelerated by some selenium-containing species [21]. However, NO-releasing material exhibits shortages such as burst release, limited storage and toxicity caused by leaching of donors. Selenium-containing catalyst organoselenium-modified polyethyleneimine (SePEI) could decompose RSNO to NO in the presence of blood through a long period of sustained and stable release. Therefore, the immobilized SePEI species on grafts could decompose RSNO to NO in the presence of blood [22].

During tissue regeneration of vascular, the inflammatory cells, especially macrophages play an important role. There are at least two different subpopulations of activated macrophages: classically activated macrophages (M1) and alternatively activated macrophages (M2) [23, 24]. M2 cells can repair tissue destruction caused by the immune response to tissue infection and secrete low levels of inflammatory cytokines and high levels of the deactivating cytokines IL-10, epidermal growth factor receptor (EGFR) ligands, vascular endothelial growth factors (VEGFs) and transforming growth factor-β (TGF-β) [18]. Moreover, M2 cells contribute to the tissue remodeling by secreting components of the extracellular matrix, including: fibronectin, osteopontin and fibrin cross-linker transglutaminase [25].

In our previous study [26], we had built a vascular graft with heparinization and *in situ* NO generation, SePEI and heparin were introduced on the surface of macroporous PCL substrates through LBL assembly. Due to the fact that nanofibers and small pores of electrospun grafts would limit cellular infiltration [27], macroporous PCL substrates were prepared in this study to facilitate cell infiltration and improve vascular regeneration. The grafts were implanted in rats to assess the histocompatibility and biological function for vascular implantation including endothelialization, IH, the proceeding of M2 polarization and tissue regeneration of the graft. We hypothesized that the SePEI/heparin-loaded material could enhance the vascular regeneration and remodeling process by mediating macrophage polarization into M2 phenotype. Our approach of combining heparinization and catalytic NO generation improves current strategies for tissue regeneration of small diameter vascular grafts.

## Materials and methods

### Materials

PCL (*M*_w_ 80 000) was purchased from Sigma (St. Louis, USA). Polydiallyldimethylammonium chloride (PDDA, *M*_w_ 100 000–200 000, 20 wt% in water) and polyethylene mine (PEI, *M*_w_ 25 000) were obtained from Sigma-Aldrich. Glutathione (GSH) was purchased from Beijing Dingguo Biotech Co. Ltd. 3-(4,5-dimethyl-2-thiazolyl)-2,5-diphenyl-2-H-tetrazolium bromide (MTT) was obtained from Lianxing Biotechnology Inc. Griess Assay Kit was obtained from Beyotime Institute of Biotechnology.

### Scaffold preparation and characterization

PCL grafts were prepared by electrospinning. Briefly, 25% (w/v) PCL solution was prepared by dissolving PCL in the mixture of methanol and chloroform (1:5 v/v), and Syringe pump (74900-05, Cole Parmer) was used for electrospinning. The collectors for the electrospun film and graft (inner diameter = 2 mm) were designed and made by our group. The electrospun fibers were collected on a rotating stainless steel mandrel with a diameter of 2 mm to form tubular scaffold. Then the scaffold was taken out from the mandrel, and dried in vacuum to evaporate the residual solvent at room temperature for 24 h.

Then the electrospun grafts were alternatively immersed into the solutions of polycation (SePEI) and polyanion (heparin) with a PDDA/heparin as a precursor layer by LBL assembly. The film was dipped into the solution (1 mg/ml) for 10 min, then rinsed with water and blown dry by air each time. The modified sample was named PCL-(SePEI/Hep)_*n*_; *n* indicated the number of layers.

As described in our previous study [26], the morphology of the electrospun films was observed using scanning electron microscope (SEM, Quanta 200, FEI, USA), the water contact angle (*n* = 10) was measured by a contact angle measurement instrument (HARKE-SPCA, China) and the catalytic NO generation was measured by Griess Assay Kit (*n* = 6).

The cytotoxicity experiment was performed in 3T3 fibroblasts (*n* = 6). Cells were cultured in a medium containing 90% Dulbecco’s modified Eagle’s medium, 10% fetal bovine serum and 1% penicillin/streptomycin at 37°C/5% CO_2_. Cells were seeded on the sample surfaces in 48-well TCPS with the density of 1 × 10^4^ per well. After 1, 3 and 5 days after seeding, the cellular compatibility was assessed by MTT test. A total of 50 μl MTT solution was added and incubated for 4 h. Then DMSO was used to replace the solution to dissolve the formazan salts. Finally the results were measured by microplate reader (Labsystems Multiskan RC) at 490 nm after incubation for 30 min at 37°C.

The electrospun films were investigated by the Fourier transformation infrared spectrometer (FTIR, TENSOR II, Bruker, Germany) in the range of 4000–500 cm^−1^. The surface chemical compositions of the specimens were measured by X-ray photoelectron spectroscopy (XPS, Axis Ultra DLD, Kratos Analytical Ltd, UK). The tensile mechanical property (*n* = 6) was determined by a universal testing machine (Instron 3345, American).

### *In vivo* implantation

Sprague-Dawley rats (male, weight 380–420 g) were operated with intraperitoneal injection of chloral hydrate (0.33 g/kg), and a single dose of heparin (100 units/kg) was administered into the tail for anticoagulation with no further anticoagulation or antiplatelet drugs. A midline laparotomy was performed, and the vascular graft (2.0 mm in inner diameter and 1.0 cm in length) was implanted for the replacement of the abdominal aorta in an end-to-end way, as described in our previous study [28]. All the rats were divided into two groups (PCL and PCL-(SePEI/Hep)_10_; *n* = 3 for each time point). The animals were sacrificed by heart perfusion of physiological saline after 1 month and 2 months respectively.

### Histological analysis and immunofluorescence staining

The grafts were explanted, rinsed with physiological saline and cut into two halves from the middle. The pieces were observed by stereomicroscopy and SEM, after then the explants were cryosectioned to 7 μm in thickness for frozen cross-section on a freezing microtome after fixed and embedded in optimal cutting temperature. The sections were stained with hematoxylin and eosin (H&E) and observed with an inverted microscope as described in our previous study [28]. To observe ECs, rabbit anti-von Willebrand factor (vWF, 1: 200, Dako, USA) was performed as primary antibody. To visualize SMCs, mouse α-SMA antibody (α-SMA, 1: 100, Boster, China) was used as primary antibody. Inflammatory cells were stained by CD68 antibody (CD68, 1:200, Abcam, USA), and M2 macrophages were stained by goat anti-human CD206 (CD206, 1:200, Santa Cruz, USA). Alexa Fluor 555 goat anti-rabbit IgG, Alexa Fluor 488 goat anti-mouse IgG (1:200, Invitrogen, USA), Alexa Fluor-594 donkey anti-mouse IgG (1:200, Invitrogen, USA) and Alexa Fluor 488 donkey anti-goat IgG (1:200, Invitrogen, USA) were used as the secondary antibodies, respectively. Sections without incubation with the primary antibody were used as negative controls. Images were observed by the fluorescence microscope (TE2000-U, Nikon Eclipse, Kanagawa, Japan).

### Statistical analysis

All quantitative results were reported as mean ± standard deviation. Student’s *t*-test was used to compare the differences. High significance was established by a value of *P *< 0.05.

## Results

### The characterization of electrospun microfibrous films

**Figure 1:**
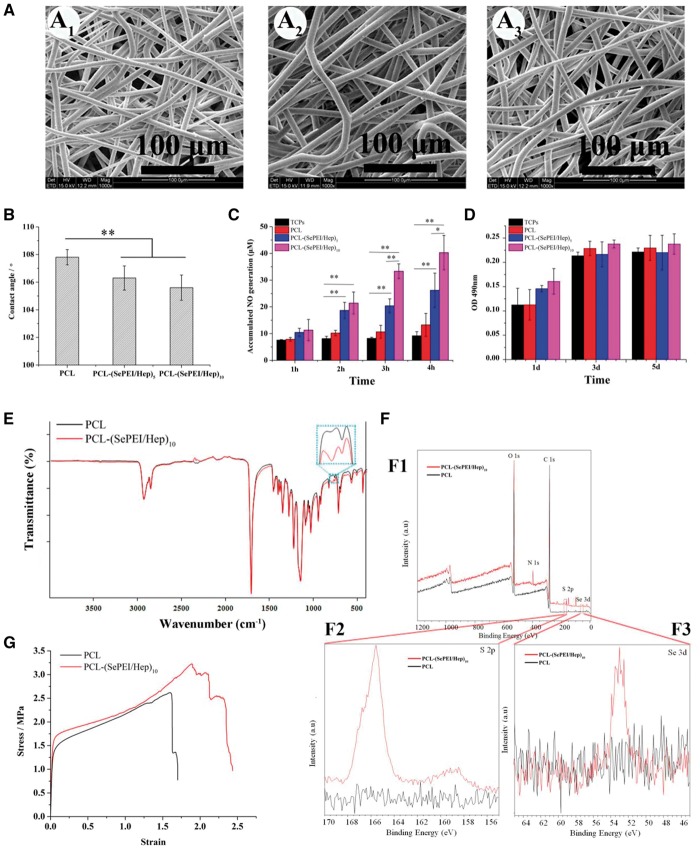
The characterization of materials. (A) SEM morphology of the electrospun fibrous films. A_1_: PCL; A_2_: PCL-(SePEI/Hep)_5_; A_3:_ PCL-(SePEI/Hep)_10_. (B) Water contact angles of the electrospun fibrous films (*n* = 10). (C) *In vitro* catalytic NO generation by films with different numbers of loaded bilayers (*n* = 6, *P < 0.05, **P < 0.01). (D) Cytocompatibility (MTT assay of fibroblast proliferation, *n* = 6). (E) FTIR spectra of the electrospun fibrous films. (F) (F1) XPS wide scans, (F2) S 2p high-resolution spectra and (F3) Se 3d high-resolution spectra of the electrospun fibrous films. (G) Stress–strain curves of the tensile mechanical property (*n* = 6)

**Figure 2:**
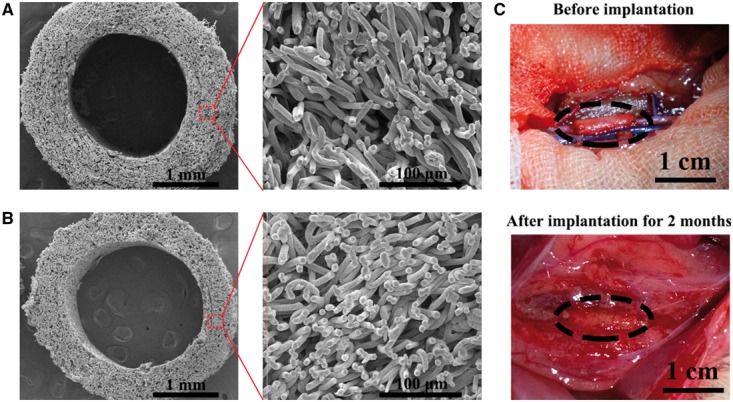
Characterization of the graft *in vivo*. (A, B) SEM images of grafts to show surface topology. (A: PCL; B: PCL-(SePEI/Hep)_10_). (C) Gross appearance of the graft before and after implantation. The oval shape (black) mark the graft location. Scale = 1 cm

The morphology of grafts exhibited smooth and bead-free fibers under SEM (Figs 1A, 2A and B). The averaged diameter was about 6.51 ± 1.02 μm for the PCL electrospun graft; while 6.41 ± 1.15 μm for the PCL-(SePEI/Hep)_10_ electrospun graft (Table 1). The outlines of the electrospun fibrous films had no significant change after surface modification. Just as many studies described, self-assembly wouldn’t affect the structural features of graft fibers [29–31].

**Table 1:** Quantitative measurement of the electrospun grafts

	Diameter (μm)	Pore size (μm)	Porosity (%)
PCL	6.51±1.02	28.41±4.49	81.05±1.38
PCL-(SePEI/Hep)_10_	6.41±1.15	27.04±1.76	80.13±1.35

The contact angle of electrospun fibrous films (Fig. 1B) decreased after assembly, which indicated that the assembly can improve its hydrophilicity slightly. The result of *in vitro* catalytic NO generation (Fig. 1C) showed that the amount of generated NO related with the layer number of assembly, which indicated that the amount of NO can be adjusted through controlling the quantities of assembled layers. In addition, there will not be a burst release in NO generation profile when this novel material contacts peripheral blood with various kinds of NO donors.

MTT test of fibroblast proliferation (Fig. 1D) showed by the cellular compatibility of materials were well. At day 1, the cell number on the PCL-(SePEI/Hep)_10_ samples was little higher than that on PCL, and at days 3 and 5, cell numbers on the PCL-(SePEI/Hep)_10_ samples were nearly the same as that on PCL.

FTIR spectra of the electrospun fibrous films (Fig. 1E) showed that after assembly, there was a characteristic peak appeared at around 802 cm^−1^. This peak may be attributed to the characteristic adsorption of S = O groups, which showed that heparin was successfully assembled [32]. XPS analysis revealed a clear S 2p signal (Fig. 1F2), also confirming the successful immobilization of heparin on the surface of PCL-(SePEI/Hep)_10_ samples; and XPS analysis revealed a Se 3d signal (Fig. 1F3), confirming the successful immobilization of SePEI on the surface of PCL-(SePEI/Hep)_10_ samples. The stress–strain curves (Fig. 1G) showed that the maximum stress of all the grafts was about 3 MPa, and the maximum strain was about 200%, while the maximum stress and the maximum strain of femoral artery were 1–2 MPa and 63–76% [33]. It suggested that all the two kinds of grafts can meet the demands of vascular tissue engineering.

**Figure 3:**
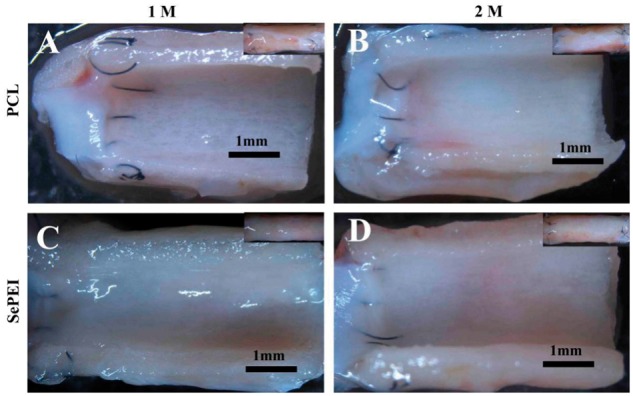
Luminal surface morphology of explanted grafts by stereomicroscope (A, C: 1 month; B, D: 2 months; A, B: PCL; C, D: PCL-(SePEI/Hep)_10_). Scale = 1 mm

### *In vivo* endothelialization

**Figure 4:**
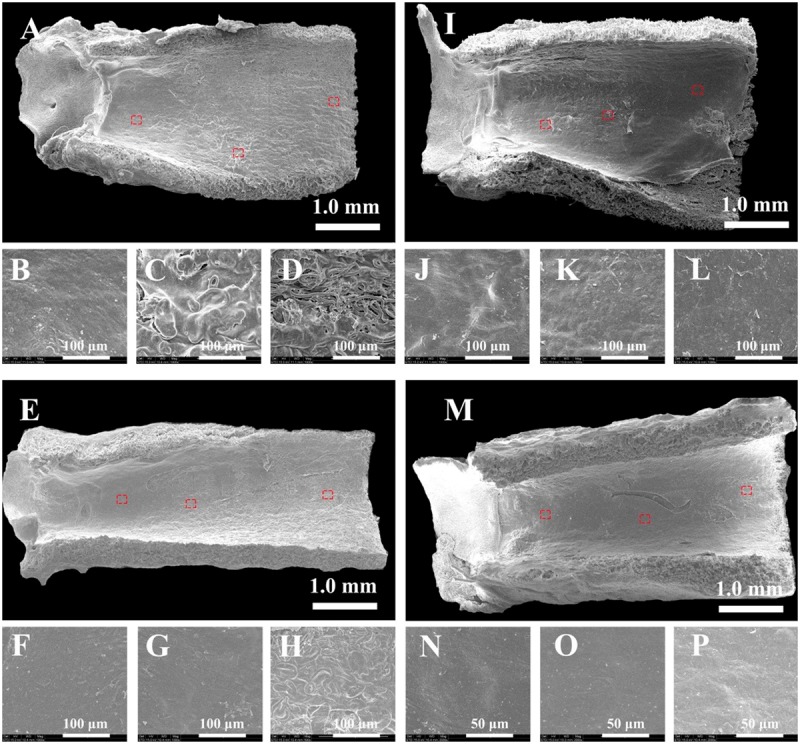
Endothelialization process of the grafts over time in rat aorta implantation observed by SEM (A–H: 1 month; I–P: 2 months; (A–D and I–L): PCL; (E–H and M–P): PCL-(SePEI/Hep)_10_). Small images are magnification of the red boxes in the corresponding large images

The grafts before implantation were observed by SEM (Fig. 2). The internal diameter of the transplanted graft was 2.0 mm, the wall thickness was about 0.6 mm and the diameter of fibers was about 6.5 μm. The cross-section of the tubular grafts demonstrated homogeneous fiber distribution, and the grafts showed large pores. Luminal surface of the grafts after implantation was observed by stereomicroscope (Fig. 3), and endothelialization of the grafts after implantation was examined by SEM (Fig. 4). Figure 3 showed that all of the grafts were patent without thrombus. After 1 month, only a small amount of endothelium was covered in the anastomosis site of PCL grafts and many fibers were obviously observed (Fig. 4A–D). In comparison, PCL-(SePEI/Hep)_10_ grafts showed more complete endothelialization and almost no fibers were observed (Fig. 4E–H). After 2 months, almost complete endothelialization of both kinds of grafts was achieved, covering the entire luminal side of grafts (Fig. 4I–P).

### Cellular infiltration

**Figure 5:**
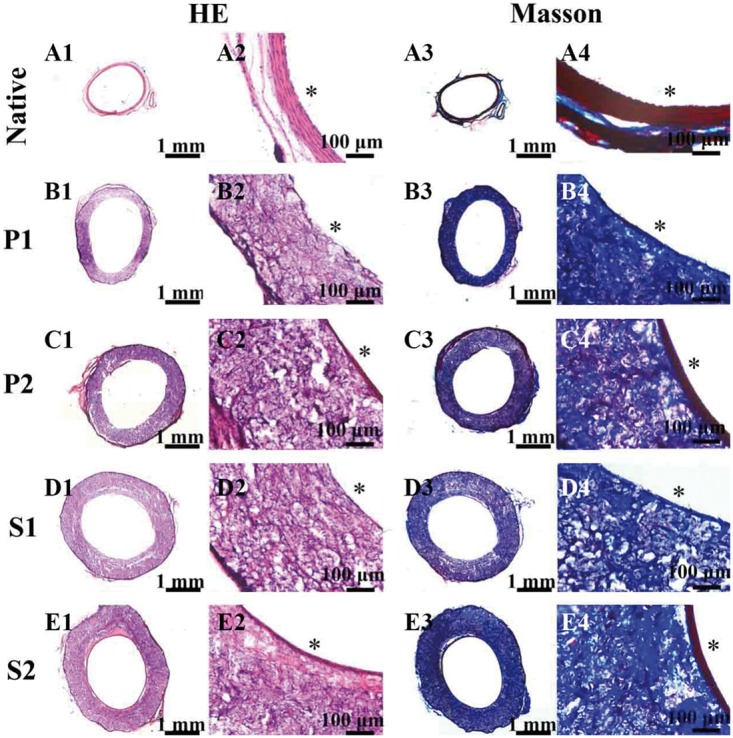
*In vivo* experiment results after 1 month and 2 months after implantation. H&E staining showed the structure of the native aorta and grafts (A1–E1, A2–E2). Masson’s trichromatic staining showed the presence of collagen (blue) in the native aorta and grafts (A3–E3, A4–E4). A1–A4: native vascular, P1 (B1–B4): PCL after 1 month, P2 (C1–C4): PCL after 2 months, S1 (D1–D4): PCL-(SePEI/Hep)_10_ after 1 month, S2 (E1–E4): PCL-(SePEI/Hep)_10_ after 2 months. Graft lumen was indicated by *

Cell infiltration was detected by H&E and Masson’s trichromatic staining. The result of H&E staining (Fig. 5) showed that cells infiltrated into the depth of the grafts and were observed within the central graft wall at all timepoints. After 1 month, the ECs on the luminal surface were continuous on PCL-(SePEI/Hep)_10_ grafts but not densely packed on PCL grafts. As shown in the result of Masson staining (Fig. 5), after 2 months, there were more amount of blue areas on PCL-(SePEI/Hep)_10_ grafts than that on PCL grafts, which indicated that the deposited collagen on PCL-(SePEI/Hep)_10_ grafts were more than that on PCL grafts. In addition, the cell layers on the lumen of PCL-(SePEI/Hep)_10_ grafts were more regular than that on PCL grafts, which were similar to the native vessel.

### Inhibition of IH

**Figure 6:**
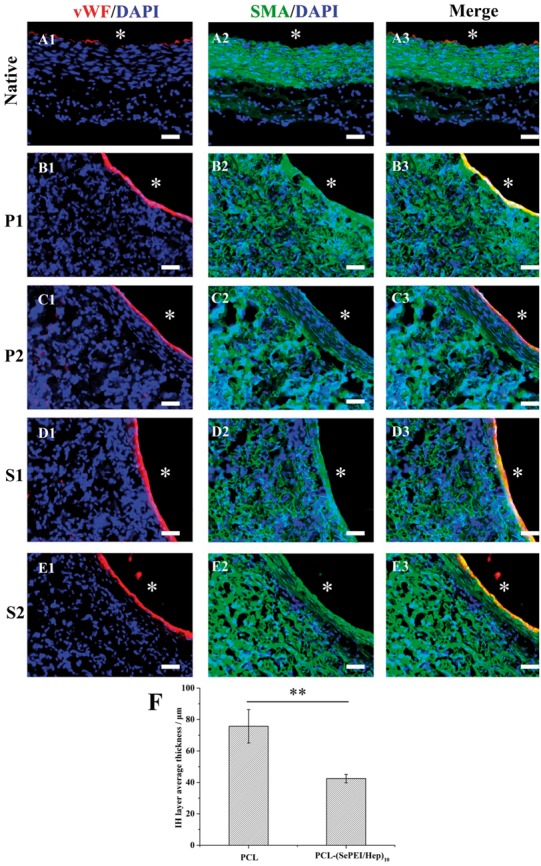
Endothelialization and smooth muscle regeneration in the grafts after 1 month and 2 months after implantation. ECs were immunostained by vWF (red), SMCs were immunostained by α-SMA (green), and cell nuclei were counterstained by DAPI (blue). A1–A3: native vascular, P1 (B1–B3): PCL after 1 month, P2 (C1–C3): PCL after 2 months, S1 (D1–D3): PCL-(SePEI/Hep)_10_ after 1 month, S2 (E1–E3): PCL-(SePEI/Hep)_10_ after 2 months. Graft lumen was indicated by *. The dotted lines indicated IH. Scale = 50 μm. (F) IH progresses after 2 months in terms of thickness is measured (**P < 0.01)

IH is a phenomenon marked by the excessive proliferation of SMCs between the endothelium and the graft [34]. As shown in Fig. 6, after 1 month, there was nearly no IH on both two kinds of grafts; while after 2 months, IH could be obviously observed on the two grafts, and the IH layer on PCL grafts (a layer of about 10 cell sheets, 75.69 ± 10.60 μm) was thicker than that on PCL-(SePEI/Hep)_10_ grafts (a layer of about five cell sheets, 42.46 ± 2.69 μm).

### Macrophage polarization

**Figure 7:**
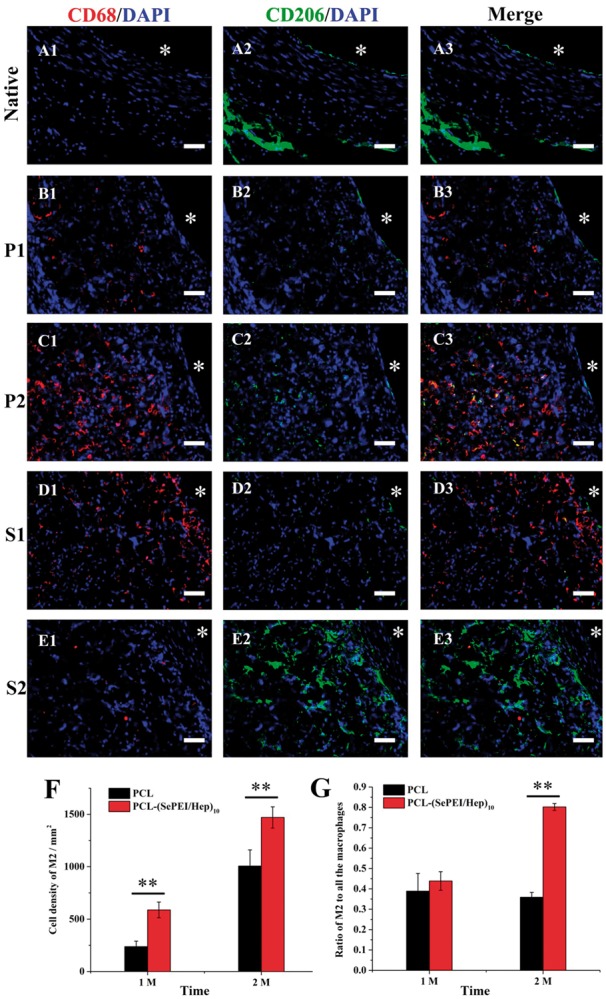
Histological assessment of the inflammation in the grafts after 1 month and 2 months after implantation. Macrophages were immunostained by CD68 (red), M2 were immunostained by CD206 (green) and cell nuclei were counterstained by DAPI (blue). A1–A3: native vascular, P1 (B1–B3): PCL after 1 month, P2 (C1–C3): PCL after 2 months, S1 (D1–D3): PCL-(SePEI/Hep)_10_ after 1 month, S2 (E1–E3): PCL-(SePEI/Hep)_10_ after 2 months. Graft lumen was indicated by *. Scale = 50 μm. (F). Cell density of M2 after 2 months. (G) The ratio of M2 to all the macrophages after 1 month and 2 months (**P < 0.01)

Macrophage polarization plays a pivotal role in the tissue regeneration and vascular homeostasis, and M2 cells are generally associated with anti-inflammatory, immunosuppressive and tissue remodeling. As shown in Fig. 7F, after 2 months, there were more M2 cells on PCL-(SePEI/Hep)_10_ grafts (Fig. 7S2) than that on PCL grafts (Fig. 7P2) and the ratio of M2 to all the macrophages on PCL-(SePEI/Hep)_10_ grafts was higher than that on PCL grafts (Fig. 7G).

## Discussion

PCL is commonly used as the main synthetic material of electrospun grafts for small diameter vascular grafts, though its lack of ability to induce cell proliferation and migration. The introduction of heparin and SePEI onto the surface of PCL would improve the cellular compatibility of grafts. Our data demonstrated that compared with PCL, the PCL-(SePEI/Hep)_10_ sample have the ability to promote the adhesion and proliferation of ECs, inhibit the proliferation of SMCs and regulate the macrophage polarization.

Previous studies have demonstrated that heparin can inhibit migration and proliferation of SMCs, demonstrating that heparin-coated grafts can help to prevent thrombosis and IH [16]. In addition, heparin can selectively enhance ECs and inhibit SMCs proliferation, which means that a heparinized surface can inhibit thrombosis and restenosis. NO can also lead to vasorelaxation and endothelial regeneration. In this study, the formation of endothelium on PCL-(SePEI/Hep)_10_ sample was clearly better than that on PCL (Fig. 4), while the thickness of the smooth muscle layer on PCL-(SePEI/Hep)_10_ sample was smaller than that on PCL (Fig. 6). It meant that the introduction of heparin improved the attachment of ECs and inhibited the proliferation of SMCs, and NO could improve this effect on the basis of heparin. These results showed that the synergistic effect of heparin and NO on selectively enhancing the attachment of ECs and inhibiting proliferation of SMCs can achieve better performance.

The foreign-body response of implanted materials is characterized by the presence of foreign-body giant cells (FBGCs) formed from the adhered macrophages. It had been shown that these multinucleated cells were responsible for the damage and failure of the implant [1]. Previous study described that the absence of endothelial nitric oxide synthase 3 (NOS3) contributes to macrophage polarization on neointimal formation. The expression of NOS3 was mediated by matrix metalloproteinases-13 (MMP-13). NO induces the expression and activity of collagenase MMP-13 [35–37], which may play a pivotal role in promoting M1 macrophages polarization to M2 macrophages, which contributed to inhibit IH [35]. Results in this study showed that the PCL-(SePEI/Hep)_10_ sample could increase the ratio of M2 macrophages (Fig. 7), which related with inhibiting IH and tissue regeneration.

In previous studies, though the cause for calcification of these grafts was not clearly understood, IH had an important role in this differentiation [34, 38]. For the long-term implantation of vascular grafts, calcification has been extensively described and more and more people have paid attention to its prevention in recent years [39]. The differentiation of SMCs into chondrocytes in the IH layers plays a crucial role in the formation of calcifications in vascular grafts. Therefore, reducing IH could improve the long-term performance of vascular grafts. In other words, the inhibition of IH can not only avoid restenosis, but also reduce calcification. In our study, PCL-(SePEI/Hep)_10_ graft obviously inhibited the thickness of IH (Fig. 6), which contributes to the decrease of restenosis and calcification. Further tests about long-term *in vivo* evaluation to assess calcification of the grafts should be conducted.

## Conclusion

In this study, we have built a vascular graft with *in situ* NO generation by introducing SePEI and heparin through LBL assembly. The graft can promote the adhesion and proliferation of ECs, inhibit the adhesion of SMCs, increase the ratio of M2 macrophages, indicated that the novel graft could enhance the vascular regeneration and remodeling process by accelerating endothelialization, inhibiting IH and mediating macrophage polarization into M2 phenotype. Further tests for longer time *in vivo* evaluation should be performed to assess the long-term patency, calcification and regenerating of the grafts. This would provide a promising method for improving tissue regeneration and long-term performance of small-diameter vascular grafts by combining heparinization and catalytic NO generation.
